# Methodological guidelines to estimate population-based health indicators using linked data and/or machine learning techniques

**DOI:** 10.1186/s13690-021-00770-6

**Published:** 2022-01-04

**Authors:** Romana Haneef, Mariken Tijhuis, Rodolphe Thiébaut, Ondřej Májek, Ivan Pristaš, Hanna Tolonen, Anne Gallay

**Affiliations:** 1 Department of Non-Communicable Diseases and Injuries, Santé Publique France, Saint-Maurice, France; 2 National Institute for Public Health and the Environment (RIVM), Bilthoven, The Netherlands; 3 Bordeaux University, Bordeaux School of Public Health, Bordeaux, France; 4 INSERM / INRIA SISTM team, Bordeaux Population health, Bordeaux, France; 5 Medical Information Department, Bordeaux University Hospital, Bordeaux, France; 6 Institute of Health Information and Statistics of the Czech Republic, Prague, Czech Republic; 7 Institute of Biostatistics and Analyses, Faculty of Medicine, Masaryk University, Brno, Czech Republic; 8 National Institute of public health, division of health informatics and biostatistics, Zagreb, Croatia; 9 Finnish Institute for Health and Welfare (THL), Helsinki, Finland

**Keywords:** Data linkage, Linked data, Machine learning techniques, Artificial intelligence, Guidelines, Methodological guidelines, Statistical techniques, Population health research, Health indicators

## Abstract

**Background:**

The capacity to use data linkage and artificial intelligence to estimate and predict health indicators varies across European countries. However, the estimation of health indicators from linked administrative data is challenging due to several reasons such as variability in data sources and data collection methods resulting in reduced interoperability at various levels and timeliness, availability of a large number of variables, lack of skills and capacity to link and analyze big data. The main objective of this study is to develop the methodological guidelines calculating population-based health indicators to guide European countries using linked data and/or machine learning (ML) techniques with new methods.

**Method:**

We have performed the following step-wise approach systematically to develop the methodological guidelines: i. Scientific literature review, ii. Identification of inspiring examples from European countries, and iii. Developing the checklist of guidelines contents.

**Results:**

We have developed the methodological guidelines, which provide a systematic approach for studies using linked data and/or ML-techniques to produce population-based health indicators. These guidelines include a detailed checklist of the following items: rationale and objective of the study (i.e., research question), study design, linked data sources, study population/sample size, study outcomes, data preparation, data analysis (i.e., statistical techniques, sensitivity analysis and potential issues during data analysis) and study limitations.

**Conclusions:**

This is the first study to develop the methodological guidelines for studies focused on population health using linked data and/or machine learning techniques. These guidelines would support researchers to adopt and develop a systematic approach for high-quality research methods. There is a need for high-quality research methodologies using more linked data and ML-techniques to develop a structured cross-disciplinary approach for improving the population health information and thereby the population health.

**Supplementary Information:**

The online version contains supplementary material available at 10.1186/s13690-021-00770-6.

## Background

The availability of data generated from different sources is increasing as well as the possibility to link these data sources with other databases. More efficient ways of data linkage and the use of artificial intelligence (i.e., machine learning techniques) are required to generate comparable and timely health information across European countries. Using these innovative techniques has several advantages such as data linkage improving completeness and comprehensiveness of information to guide health policy processes [1]. New approaches more or less based on artificial intelligence allow us to handle data with a large number of dimensions (features) and units (feature vectors) more efficiently and with high precision. Many countries have already invested in the linkage including both deterministic and probabilistic linkages and linking their traditional health administrative data with other types of data and has increased interoperability [2]. The capacity to use data linkage and artificial intelligence (AI) to estimate and predict health indicators varies across European countries [3]. However, the estimation of health indicators from linked administrative data is challenging due to several reasons such as variability in data sources and data collection methods, interoperability issues (legal, organizational, semantic and technical levels), availability of a large number of variables, lack of skills and capacity to link and analyze big data [4]. Due to varying health information systems across European countries, makes challenging to learn from each other experiences.

To our knowledge, there are no methodological guidelines available, which could systematically guide countries in using linked data and/or machine learning techniques (ML-techniques) to estimate health indicators for population health research and monitoring. Therefore, the InfAct project has proposed to develop these guidelines, which could guide those MSs who are planning to estimate health indicators using linked data and/or ML-techniques with new methods/techniques. InfAct (Information for Action) project is a joint action of Member States (MSs) aiming to develop a more sustainable EU (European Union) - health information system through improving the availability of comparable, robust and policy-relevant health status data and health system performance information [5]. InfAct gathers 40 national health authorities from 28 MSs.

The main objective of this study was to develop the methodological guidelines to estimate population-based health indicators using linked data and/or ML-techniques with new methods.

## Methodology

We have performed following step-wise approach systematically to develop the methodological guidelines: i. scientific literature review, ii. identification of inspiring examples from European countries and iii. Developing the checklist of guidelines contents.

### Literature review

Firstly, we performed a literature search to identify published articles focusing on estimating health indicators using linked data and/or machine learning techniques in the field of health surveillance and health care performance on August 1, 2020. We did not specify any time period to search for the related published articles as to obtain a wide range of studies published at any time. We included in our search peer-reviewed methodological articles, related guidelines and systematic reviews that were published in the English language. We excluded those studies published as protocols, scoping reviews or literature reviews, non-methodological studies such as editorials, commentary or perspectives and studies related to life sciences such as RNAi or gene expression. We defined two search strategies to extract the citations from Pubmed with following keywords: search strategy 1 (Linked data, Machine learning techniques and Guidelines) and search strategy 2 (Health indicators, Linked data, Machine learning techniques and Guidelines). Further details of search strategies are reported in additional file 1. Based on this literature review, we identified various methodological approaches using linked data and/or machine learning techniques to develop these guidelines.

### Identifying inspiring examples

We defined inspiring examples as those studies that take into account the use of linked data and/or ML-techniques to estimate health indicators and implied the estimated health indicators to target priority public health actions (i.e., surveillance, prevention, promotion, etc.), healthcare strategies or to guide/support public health policies according to their geographical regions. We asked ten European countries who were part of InfAct project and have been performed studies using linked data and/or machine learning techniques [6].

### Developing the checklist of methodological guidelines contents

Using the results of first two steps, we reviewed the method section of selected studies and have developed a checklist including the following items for guidelines: rationale and objective of the study (i.e., research question), study design, linked data sources, study population/sample size, study outcomes, data preparation, data analysis and study limitations.

#### Expected outcomes

The methodological guidelines to estimate health indicators focused on population health research using linked data and/or ML-techniques.

## Results

### Literature review

**Fig. 1:**
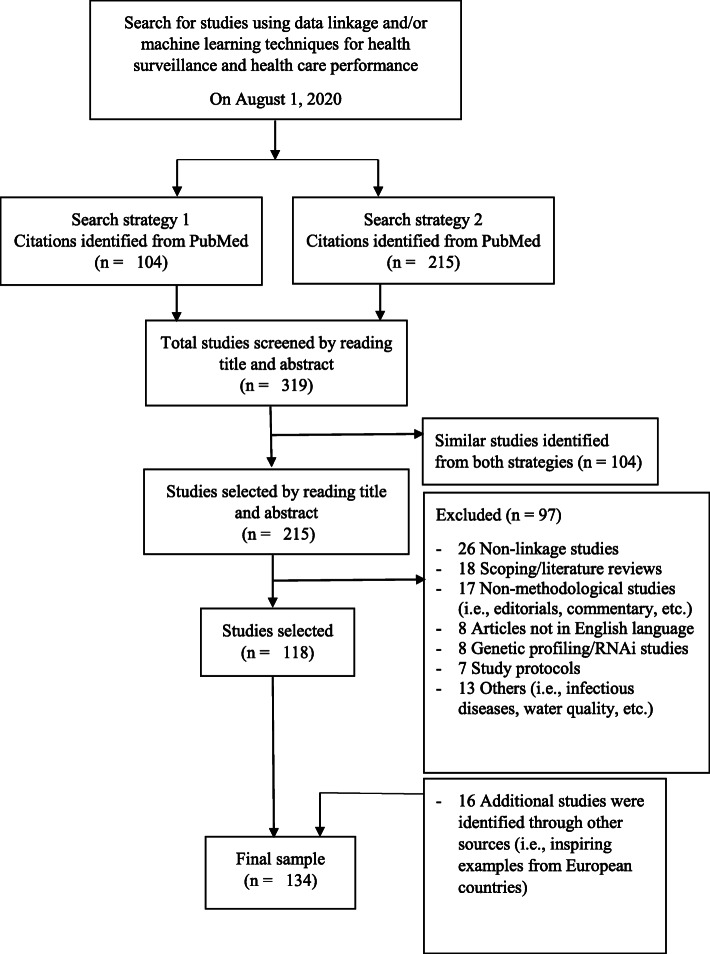
Flow diagram of search strategy used to identify studies using data linkage and/or machine learning techniques for health surveillance and health care performance to develop the methodological guidelines to estimate population-based indicators, a study performed under InfAct project, May 2021

We reviewed 215 citations from PubMed and 118 were included in our final sample to develop these methodological guidelines (Fig. 1). Sixteen additional studies (i.e., inspiring examples from European countries) identified from InfAct project were also included to the final sample. The final sample included 134 studies using linked data and/or machine learning techniques to address various research questions either to describe or estimate health indicators in the field of health status monitoring or the evaluation of certain treatments in medical/health care. Among these citations, some guidelines were also identified to adopt the appropriate format of methodological guidelines [7, 8]. We reviewed the methodologies applied in the selected studies and developed a check-list of various steps that could be adopted systematically to calculate health indicators using linked data and/or ML-techniques.

### Inspiring examples

We have identified 16 studies as inspiring examples from ten European countries. These studies adopted various methodological approaches to estimate health indictors, either by using data linkage (12 studies), machine learning methods (2 studies) or both data linkage and machine learning approaches (2 studies). These studies were used to develop these guidelines.

### Methodological guidelines for studies using linked data and/or ML-techniques

We have developed a checklist of key methodological steps that are recommended to adopt systematically while calculating population-based health indicators (Table 1) and include the following items as methodological guidelines with examples of studies:

**Table 1:** Methodological guidelines using linked data and/or machine learning techniques to estimate population-based indicators, a study performed under InfAct project, May 2021

Item number	Checklist item	Description	
**1**	**Rationale and objective of the study (i.e., research question)**	Define the rationale and objective of the study by adopting PICO criteria to research studies focused on population health.	☐
**2**	**Study design**	Select the appropriate study design that could best address the proposed research question.	☐
**3**	**Linked data sources**	Select the required linked data sources to answer the proposed research question.	☐
**4**	**Study population**		
4.1		Define the inclusion and exclusion criteria of the study population by taking into account age, sex and period of data collection.	☐
4.2	Sample size	State the significance level of alpha and power based on the defined research question to calculate the sample size.	☐
**5**	**Study outcomes**		
5.1	Main outcomes	Define the main outcomes by taking into account study population, health condition to be studied, exposure (intervention/risk factors, if relevant) and defined period of study.	☐
5.2	Level of estimation	Describe the level of estimation of health outcomes at the lowest possible granularity level (i.e., at community, metropolitan, departmental or regional levels).	☐
**6**	**Data preparation**		
6.1	A. Data extraction	Extract data with required input variables from linked data set to a single file or a spreadsheet that could be converted according to the required format of the statistical software for data analysis.	☐
6.2	Coding of variables	Code the input variables, which are common in different linked data sets continuous or categorical or binary variables for required data analysis.	☐
	B. Data preparation to develop and apply a ML-algorithm		
6.3		Identify and define the target groups for a given defined time window based on the outcome of interest.	☐
6.4		Code the inputs variables, which are common in different linked data sets to continuous or categorical or binary variables for a given defined time window time.	☐
6.4		Split of final data set into 80% training and 20% test data set.	☐
**7**	**Data analysis**		
7.1	A. Variables selection	Select variables after the removal of all variables with a variance equal to zero.	☐
7.2		Estimate the RelifExp score based on the relevance of each variable to the outcome of interest.	☐
	B. Statistical techniques		
7.3	I. Classical statistical techniques	Select an appropriate statistical technique to address the proposed research question according to the study objectives and the available data.	☐
	II. ML-techniques		
7.4		Train various models and compare the performances of each model in terms of AUC curve (only for binary classifier).	☐
7.5		Validate the model performance using k-fold cross-validation first on training data set, and then assess the model performance on test data set.	☐
7.6		Select the final model based on specific performance metrics including sensitivity, specificity, PPV*, NPV^*^, F1-score and kappa.	☐
	C. Sensitivity/uncertainty analysis		
7.7		Perform a sensitivity analysis to identify the most influential parameters for a given output of a model.	☐
7.8		Select an appropriate method to perform the sensitivity analysis.	☐
7.9		Calculate the uncertainty in estimates using 95% CI^*^ and describe the source of uncertainty (if relevant).	☐
	D. Potential issues during data analysis		
	I. Missing data		
7.10		Identify the missing data in the given dataset.	☐
7.11		Apply an appropriate technique for the imputation of missing values in the given data set.	☐
7.12	II. Imbalanced target group in a given dataset	Apply an appropriate technique to create a balanced data set either using down sampling or over sampling approach.	☐
7.13	III. Bias and variance tradeoff	Find the most generalizable model to keep the balance between bias and variance.	☐
**8**	**Study limitations**	Describe the study limitations related to data sources (i.e., linkage, quality, access and privacy), study design, study population and statistical method used (if relevant).	☐

*PPV Positive Predictive Value, *NPV* Negative Predictive Value, *CI* Confidence interval

#### Rationale and objective of the study (i.e., research question)

The first step is to define the research question for the proposed study. The PICO criteria (P = Population/patient problem, I = Intervention/exposure/risk factor, C = Comparator/control/alternative intervention [if appropriate], O = Outcome) are used in evidence-based practice to frame and answer clinical and health care-related questions [9]. These criteria could be adopted according to population health research questions. The research questions should be simple and smart for example “for obese children (population), does the use of community recreation activities (intervention) compared to educational programs on lifestyle changes (comparator) reduce the risk of diabetes mellitus (outcome)? The population based studies commonly focused on estimating the health indicators, associations between health outcomes and exposures, identifying health inequalities, predicting the health indicators/outcomes, classifying population groups to estimating their health outcomes, etc.

#### Study design

The second step is to select the appropriate study design that could best address the proposed research question. The choice of study design may depend on the type of available data. Following were the most commonly used study designs (see additional file 2): cross-sectional studies (for estimating the associations between health outcomes and various exposures); population-based cohort (for estimating and predicting health outcomes [e.g. incidence/prevalence] in context of certain risk factors, disease care, classifying population groups to estimating their health outcomes); and a case-control studies (for comparing exposure between cases and controls), etc.

#### Linked data sources

The third step is to select the required linked data sources or one large data source to answer the proposed research question with specific objective. The linked data sources added the value and complement information on various factors which may have direct or indirect influence on health indicators. The health administrative data sources (i.e., hospital discharge, mortality, primary care/general practitioners, health insurance claims), which are either linked with each other or with other data sources (i.e., disease-specific registries, health surveys, epidemiological cohort studies, vital statistics), are the most commonly used data sources. These data sources are linked using both deterministic and probabilistic data linkage techniques.

#### Study population/sample size

The fourth step is to define the study population according to the proposed research question. Often, the study population is extracted from the national health administrative database linked either with a population-based cohort or disease-specific registry or health survey or with any other administrative database. The linked database may allow having a large sample size and adds to large number of variables for analysis. The large sample size allows stratified analysis among sub-groups. The inclusion and exclusion criteria of the study population should be clearly defined according to the research question. The age, sex of included sample and the period of data collection should be clearly stated.

The null and alternative hypothesis should be clearly stated based on the research question [10]. The commonly used statistical significance values of alpha are 0.01, 0.05 or 0.1. To calculate the sample size, significance level of alpha and power should be stated based on the defined research question.

#### Study outcomes and their estimation at various geographical levels

The fifth step is to define the study outcomes according to the proposed research question. The study outcomes should be clearly defined by taking into account the study population, health condition (to be studied), exposure (intervention or risk factors if relevant) and the defined period of study. The PICO criteria could also be used to define the study outcomes [9].

It is important to estimate the health outcomes at the lowest granularity level (i.e., at the community, metropolitan, departmental or at regional levels) to highlight the variability at various geographical level and to adopt the health decisions according to the local needs.

#### Data preparation

The six step involve data preparation with two possibilities: raw data extraction and developing and applying ML-algorithm (Table 1).

##### Raw data extraction

This step involves raw data extraction with required input variables from the linked data sets without applying ML-techniques. The extracted data from linked sources could be exported to a single data file or a spreadsheet that could be converted to different file formats according to the statistical software to be used for data analysis. The input variables, which are common in different linked data sets could be coded to binary or continuous or categorical variables. The dates are coded as a continuous variable. The linked data could add some redundancies while linking some variables to extract one specific information and to perform specific analyses.

##### To develop and apply a ML-algorithm

This step involves the preparation of data to develop and apply an ML-algorithm, following sub-steps:

I. *Target/Case definition:* First, the targets are identified and defined based on the outcome of interest either as positive target (cases, for example, pharmacologically treated diabetes patients) or as negative target (controls, for example, non-diabetes patients) for a given time window (e.g., pharmacologically treated diabetes patients in last 6 months are defined as positive targets and non-diabetes patients in last 6 months are defined as negative targets).
II. *Coding of variables for a given time window:* All the input variables, which are common in different linked data sources, are coded to binary or continuous or categorical variables for a given defined time window (e.g., either 6 or 12 months). The choice of a time window to code is important and should be selected based on the research question and study objectives.
III. *Split of final data into training and test data sets:* In most of the studies, the final data set is split into 80% as a training data set and 20% as a test data set. If there is an imbalance of number of positive target - 1 group over the number of negative target - 0 group in the training dataset, a random down sampling or over sampling can be performed in the target 0 group to achieve the same number of individuals in both target groups. This helps to avoid the bias in ML-algorithm. Later, the selection of variables and the models is performed using the training data. The test data is used solely to test the final model performance.
  According to the context of study and available data, different techniques of normalizing the data and cross-validation could be used.

#### Data analysis

The seventh step is the data analysis that may include variable selection, application of different statistical techniques, sensitivity/uncertainty analysis and some potential issues that may encounter during the data analysis (Table 1).

##### Variables selection

First, all variables with a variance equal to zero are removed. Then the ReliefF exp. method could be applied (i.e., is a noise tolerant method and is not affected by features interactions) to estimate the score based on the relevance of each variable to the outcome of interest and to minimize the collinearity effect [11]. All variables are ranked according to the ReliefF exp. score and for continuous variables the score ranges from 0 to 1. For example, the cutoff score could be selected based on the visual inspection of the ordered plot of ReliefF values for all variables, called “elbow plot” approach (e.g., 0.01). In this case, the variables that had a ReliefF exp. score equal or more than 0.01 could be included to train different models and the variables less than 0.01 could be excluded.

##### Statistical techniques

There are several statistical techniques that are applied to linked data either using classical statistical techniques or with ML-techniques. The former may be used for regression and later for classification purposes. In general, both of these techniques could be used to estimate, classify and predict the population health indicators or to evaluate the health care interventions according to the available linked datasets. The brief description of different techniques is reported in additional file 2.

I. ***Classical statistical techniques:*** Several classical statistical techniques were identified in the selected studies to analyze the linked data set. Following are the most commonly used techniques: linear and logistic regression, Linear Discriminant Analysis (LDA) model [12, 13], multilevel linear regression [14], multivariate logistic regression [15], multivariable hierarchical modified Poisson regression [16], Cox regression models [17], LASSO regression [18, 19], Generalized Estimating Equation (GEE) models [20], inverse probability weighting methods [21], Blinder-Oaxaca decomposition method [22] and Markov modelling [23].
II. ***ML-techniques:*** Several ML-techniques are applied, which focused on health care research. These techniques could be adopted to population health studies. Following are the most commonly used supervised ML-techniques: linear and logistic regression, Linear Discriminant Analysis (LDA) model [12, 13], partial least square discriminant analysis model [24], decision tree [25], random forest [26] and Gradient Boosting Classifier [GBC] [27, 28], k-nearest neighbours/k-means [29], support vector machine [SVM] [30], neural networks [31], convolutional neural networks, hierarchical clustering [32] and XGBoost [33].

To develop and apply ML-techniques, following three main steps are used to train and select the final model:

- *Training various models:* Some commonly used models are linear discriminant analysis, logistic regression, flexible discriminant analysis and decision trees that are applied to the training data set. The performance of each model is compared in terms of area under the receiver operating characteristic (AUC) curve. AUC curve is an evaluation metric for binary classification problems. It is a probability curve that plots TPR (true positive rates) or sensitivity against FPR (false positive rates) or I – Specificity at various threshold values and essential separates the ‘signal’ from the ‘noise’. The AUC is the measure of the ability of a classifier to distinguish between classes [34]. The higher the AUC, the better the performance of the model at distinguishing between positive and negative classes.
- *Model validation techniques:* To validate the model, k-fold cross-validation is commonly used technique. Using this technique, the given data set is split into a *K* number of sections/folds where each fold is used as a testing set at some point. For example, 5-fold cross validation (K = 5) where the data set is split into 5 folds. In the first iteration, the first fold is used to test the model and the rest are used to train the model. In the second iteration, 2nd fold is used as the testing set while the rest serve as the training set. This process is repeated until each fold of the 5 folds have been used as the testing set [35]. This technique allows to estimate the performance or accuracy of the model using data not utilized during training of the model.

After the first validation of the models using k-fold cross-validation on training data set, the model performances are assessed using the test data set.

iii. *Selection of final model*: After the model validation, the algorithm selection process is automated by giving the computer a specific metrics including sensitivity, specificity, positive predictive value, negative predictive value, F1-score and kappa. Finally, a single model is retained based on its performance, computational parsimony and its transferability to other databases.

##### Sensitivity/uncertainty analysis

After the selection of final model, sensitivity analysis is performed. This analysis refers to identifying the most influential assumptions or parameters for a given output of a mathematical computer model (i.e., the sensitivity of output by changing the inputs) or to evaluate the effect of uncertainty in each uncertain computer input variable on a particular model output [36]. It helps to understand the relationship between input and output variables and the robustness of the results of a computing model [37]. The most common methods are: variance-based method [38], elementary effects method [39] and regression analysis.

##### Potential issues during data analysis

During the data analysis, following are some common issues, which may encounter: missing data, imbalanced datasets and bias-variance tradeoff.

I. ***Missing data:*** In datasets (small or big), missing values are often the main issue that can introduce a substantial amount of bias, make handling and data analysis harder and strongly influence the model performance.

There are three types of missing data [40]: 1. Missing Completely At Random (MCAR): if subjects who have missing data are a random subset of the complete sample of subjects, 2. Missing Not At Random (MNAR): if the probability that an observation is missing depends on information that is not observed, like the value of the observation itself is missing, and 3. Missing At Random (MAR): the probability that an observation is missing commonly depends on information for that subject that is present i.e., the reason for missing data is based on other observed patient characteristics.

***Imputations of missing values:*** Imputation is a process of replacing missing values in a dataset. Following are some common approaches, which could be applied to both type of studies using classical statistical methods and ML-techniques:

- ***For classical statistical methods:*** There are three most commonly used techniques i.e., 1. listwise/complete case deletion, 2. single imputation and 3. multiple imputations. Simple/single imputation techniques (e.g. linear regression) for handling missing data (such as complete case analysis, overall mean/mode/median imputation, and the missing-indicator method) are more feasible to apply but may produce biased results. Multivariate Imputation by Chained Equation (MICE) is a multiple imputation techniques and does not avoid all bias but may be less prone to bias and does not help with MNAR [40, 41].
- ***For ML-studies:*** There are eight most common ways to replace the missing values, which could be applied in both non-ML and ML-models: 1. rows/listwise/complete case deletion, 2. replacing with mean/median/mode, 3. assigning a unique category, 4. using most frequent or zero/constant values, 5. predicting the missing values using linear regression, 6. using algorithms which support missing values, 7. Multivariate Imputation by Chained Equation (MICE) and 8. deep learning (DataWig) [42, 43]. These techniques are also robust to MAR data.
  Instead of data imputation, a novel method based on additive least square support vector machine (LS-SVM) is potentially a promising technique for tackling missing data in epidemiological studies and community health research [44].

II. ***Imbalanced datasets:*** Second issue is the imbalanced dataset (i.e., the number of positive and negative targets/cases/values are unequal.) that can skew in class distribution and may bias ML-algorithms. Many ML-techniques, such as neural networks, make more reliable predictions from being trained with balanced data [45]. There are two commonly used approaches to create a balanced data set, first is the down sampling and the second one is over sampling [45, 46].
III. ***Bias and variance tradeoff:*** The third issue is the bias and variance tradeoff. The concept of bias and variance and their relationship with each other is fundamental to the true performance of supervised ML models [47]. Bias refers to the error in the ML-model due to wrong assumptions. A high-bias model will underfit the training data. Variance refers to problems caused due to overfitting. This is a result of the over-sensitivity of the model to small variations in the training data. A model with many degrees of freedom (such as a high-degree polynomial model) is likely to have high variance and thus overfit the training data. Increasing a model’s complexity will reduce its bias and increase its variance. This is also the rational for cross-validation approaches. This balance is key to finding the most generalizable model [47].

***Model tuning/hyperparameter tuning:*** It is an important step to improve the model performance and accuracy. Robust model tuning provides insight on how model structure and hyperparameters influence the model performance [48]. Hyperparameters a**r**e adjustable parameters that must be tuned in order to obtain a model with optimal performance. There are some techniques, which are commonly used to tune the hyperparameters: grid search, random search and Bayesian optimization [49].

#### Study limitations

Study limitations are important and should be reported to addressing various issues for further research. Different studies using data linkage and/or ML-techniques reported some common study limitations related to data sources (linkage, quality, access and privacy), study design and statistical methods. Following are some limitations, which may influence the quality of research studies: ***Data linkage*** (e.g., different data collection methods in different areas make it difficult to link and to compare the data, lack of standard methods for data collection or inability to link some cases due to incorrect identifier); ***Data quality*** (e.g., lacking completeness of information for some routinely collected data sources, unavailability of certain information to improve the results of some analyses, lacking information on secondary cause of death, exclusion of some groups for whom no linkage could be done due to lack of identifier); ***Access/availability of certain data sources*** (e.g., readily unavailability/inaccessibility of data related to employment, education, occupation and socioeconomic status, lack of data on health inequalities at local levels); ***Data privacy*** (e.g., certain variables cannot be explored due to privacy or confidentiality issues, legal interoperability issues to link various data sources); ***Study design*** (e.g., causality, misclassification of exposure outcome, bias, age of study sample, use of isotropic model of exposure)***; Study methods*** (e.g., appropriate choice of a time window to code the variables to estimate the incidence, overfit or underfit of the model used in ML-studies, boosted algorithm may require a high computational capacity).

## Discussion

### Main results

We have developed a checklist of eight items as the methodological guidelines, which provide a systematic approach using linked data and/or machine learning techniques to produce population-based health indicators.

There are few studies available that describe the reporting guidelines for linked data focused on population health research. For example, one study illustrates the guidelines to evaluate the methodological quality of studies using linked data and to report their results in a consistent manner [8]. Another study defines the best reporting practices as guidelines for accurate and transparent reporting of health estimates for studies that calculate health estimates for multiple populations (in time or space) using multiple information sources [7]. Another study developed TIDieR-PHP (Template for Intervention Description and Replication-Population Health and Policy) checklist to improve the reporting of PHP (population health and policy) interventions [50]. The STROBE (Strengthening the Reporting of Observational Studies in Epidemiology) guidelines were developed for reporting of observational studies [51]. The STRATOS (STRengthening analytical thinking for observational studies) initiative was taken to provide accessible and accurate guidance in the design and analysis of observational studies in medical research [52]. These guidelines could also be used for population health research studies. All these guidelines are important to improve the design, analysis and reporting of results. Nevertheless, the existing reporting guidelines are not fully designed to capture key methodological aspects applied to linked data and/or ML techniques for population health research.

### Scope

These guidelines define a systematic approach for studies using linked data and/or ML-techniques to estimate health indicators for population health research. We used peer-reviewed published methodological studies, which applied data linkage and ML-techniques in the field of health status monitoring and medical/health care for the estimation and prediction of health indicators. These guidelines offer a general framework of methods to be used for the calculation of health indicators and are flexible enough to integrate new methods used for population health research over time.

### Implications

These guidelines would assist public health researchers and epidemiologists to develop and adopt new methods/techniques using linked data and machine learning approaches for their studies. These guidelines would also allow to harmonize and practice certain methodological approaches to perform comparative studies between countries. Moreover, these would add to high-quality evidence-based research to guide health policy decisions.

### Strengths and limitations

This is the first study to develop methodological guidelines with a systematic approach to perform studies using data linkage and/or machine learning techniques to calculate health indicators for population health research. Moreover, these guidelines would improving the quality of research methods. We have provided at least one example of a study that has used the reported statistical techniques to better understand different aspects.

There are few limitations: *first,* we provided a systematic approach with general and basic techniques that are most commonly applied for studies using data linkage and/or ML-techniques. More techniques are possible, which are not reported here and could be applied to answer various research questions to improve the population health research. However, the reported techniques cover the main and basic techniques, which are commonly applied. *Second,* there are more studies possible, which have applied these techniques and are not reported in this study. Though the studies which are reported here, have covered the important techniques. *Third,* we did not perform a systematic review to identify the studies. Nevertheless, the adopted search strategy allowed to identify relevant studies and covered all the basic aspects of studies.

### Recommendations

We proposed the following recommendations that not only address some of the study limitations identified but also promote the population-based research studies using linked data and/or ML-techniques:

*Data sources:* data related to employment, education, occupation and socioeconomic status should be readily available/accessible to enrich the analyses related to the health status, standard methods for data collection should be implemented in a health information system and routinely data collected from various administrative sources should improve their quality concerning to the completeness of the information. *Data regulations:* specific mandates to ensure data availability/access/capture and safe storage should be an integral part of a national/regional health information system, differences in the implementation and interpretation of the EU-GDPR (General Data Protection Regulations) and additional national regulations should be mapped and if possible harmonize the implementation of GDPR across EU-MSs [53]. *Study design:* the rational selection of the study design using linked data is important to avoid certain methodological limitations. *Statistical methods:* the use of an appropriate statistical technique is important to have results that are more robust. *Knowledge translation*: better approaches are required to communicate the estimates to the policymakers and other public stakeholders. This is key to evidence-informed policymaking and to support decision making about the allocation of resources. *Collaborations:* more collaborations among the Member States for an exchange of inspiring examples/best practices in using linked data and machine-learning approaches are needed. Moreover, to develop joint country studies among European countries on using machine-learning techniques for public health research are needed.

## Conclusions

This is the first study to develop the methodological guidelines to estimate population-based health indicators using linked data and/or machine learning techniques. These guidelines would support researchers to adopt a systematic approach with high-quality research methods. Using linked data and ML-techniques have the potential to add value in research focused on population health. However, the overall generalizability of ML-models in real-world data is critical and the researchers should aware of their data limitations. There is a need for high-quality research methods using more linked data and ML-techniques to develop a structured cross-disciplinary approach for improving the population health information and thereby the population health.

## Supplementary Information

**Additional file 1.** It describes the search strategies used to identify citations related to data linkage and/or machine learning technique used for studies focused on health status monitoring and health care.

**Additional file 2.** It describes the various statistical techniques used for data analysis using both classical statistical techniques and ML-techniques.

## Data Availability

Not applicable.

## Abbreviations

EU: European Union
MSs: Member States
AI: Artificial Intelligence
ML-techniques: Machine Learning Techniques
InfAct: Information for Action i.e., a joint action of Member States to establish a sustainable European health information system
PICO Criteria: Population-Intervention-Comparator-Outcome Criteria
LASSO: Least Absolute Shrinkage and Selection Operator
GEE: Generalized Estimating Equation
GBC: Gradient Boosting Classifier
SVM: Support Vector Machine
LDA: Linear Discriminant Analysis model
FDA: Flexible Discriminant Analysis model
XGBoost: Extreme Gradient Boosting
HWNNs: Hybrid Wavelet Neural Networks
SOM: Self-Organizing Maps
ROC: Receiver Operating Characteristics
MCAR: Missing Completely At Random
MNAR: Missing Not At Random
MAR: Missing At Random
MICE: Multivariate Imputation by Chain Eq.
LS-SVM: Lease Square-Support Vector Machine
PPV: Positive Predictive Value
NPV: Negative Predictive Value
GDPR: General Data Protection Regulations
