# Correction to: Methodological guidelines to estimate population-based health indicators using linked data and/or machine learning techniques

**DOI:** 10.1186/s13690-022-00831-4

**Published:** 2022-02-23

**Authors:** Romana Haneef, Mariken Tijhuis, Rodolphe Thiébaut, Ondřej Májek, Ivan Pristaš, Hanna Tolonen, Anne Gallay

**Affiliations:** 1 Department of Non-Communicable Diseases and Injuries, Santé Publique France, Saint-Maurice, France; 2 National Institute for Public Health and the Environment (RIVM), Bilthoven, The Netherlands; 3 Bordeaux University, Bordeaux School of Public Health, Bordeaux, France; 4 INSERM / INRIA SISTM team, Bordeaux Population Health, Bordeaux, France; 5 Medical Information Department, Bordeaux University Hospital, Bordeaux, France; 6 Institute of Health Information and Statistics of the Czech Republic, Prague, Czech Republic; 7 Institute of Biostatistics and Analyses, Faculty of Medicine, Masaryk University, Brno, Czech Republic; 8 National Institute of Public Health, Division of Health Informatics and Biostatistics, Zagreb, Croatia; 9 Finnish Institute for Health and Welfare (THL), Helsinki, Finland

**Correction to: Arch Public Health 80, 9 (2022)**

**https://doi.org/10.1186/s13690-021-00770-6**

Following publication of the original article [1], the authors reported that the sixth author's family name was incorrectly spelled as “Tolenan”.

The correct spelling of the family name “Tolonen” has been provided in this Correction.

The original article [1] has been updated.

## References

[1] Haneef R, Tijhuis M, Thiébaut R (2022). Methodological guidelines to estimate population-based health indicators using linked data and/or machine learning techniques. Arch Public Health.

