# Supplementary material for: Methodological guidelines to estimate population-based health indicators using linked data and/or machine learning techniques
Source: Arch Public Health. 2022 Jan 4;80:9. doi: 10.1186/s13690-021-00770-6 (PMC8725299; doi:10.1186/s13690-021-00770-6)
Supplement: Supplementary file 1 — Additional file 1. It describes the search strategies used to identify citations related to data linkage and/or machine learning technique used for studies focused on health status monitoring and health care. [file 13690_2021_770_MOESM1_ESM.docx]

**Additional file 1: Search strategies performed on August 1, 2020 to extract citations from Pubmed**

*Search strategy 1*: ((Linked data [Title/Abstract] OR Machine learning techniques [Title/Abstract]) AND Guidelines [Title/Abstract]))

*Search strategy 2*: ((Health indicators [Title/Abstract] OR Linked data [Title/Abstract]) OR Machine learning techniques [Title/Abstract]) AND Guidelines [Title/Abstract]))
