# Supplementary material for: Methodological guidelines to estimate population-based health indicators using linked data and/or machine learning techniques
Source: Arch Public Health. 2022 Jan 4;80:9. doi: 10.1186/s13690-021-00770-6 (PMC8725299; doi:10.1186/s13690-021-00770-6)
Supplement: Supplementary file 2 — Additional file 2. It describes the various statistical techniques used for data analysis using both classical statistical techniques and ML-techniques. [file 13690_2021_770_MOESM2_ESM.docx]

**Additional file 2: Brief description of statistical techniques used in various studies**

We have identified 19 different statistical techniques used in various studies either for health monitoring or to improve medical or health care. More techniques are possible to apply. However, here we describe the brief description of some models used in various studies under two categories: 1. Classical statistical techniques and 2. Machine learning techniques.

**1. Classical statistical techniques (without applying machine-learning techniques) (N = 9)**

1. **Multilevel, multiple linear regression models**

*Model description:* Multiple linear regression (MLR), also known simply as multiple regression, is a statistical technique that uses several explanatory variables to predict the outcome of a response variable. The goal of multiple linear regression (MLR) is to model the [linear relationship](https://www.investopedia.com/terms/l/linearrelationship.asp) between the explanatory (independent) variables and response (dependent) variable.

*Title of the study:* Associations between fast food and physical activity environments and adiposity in mid-life: cross-sectional, observational evidence from UK Biobank

*Link to the study:* <https://doi.org/10.1016/S2468-2667(17)30212-8>

*Study design:* Cross-sectional study

*Domain:* Health status monitoring

*Data sources used:* Population based cohort linked with spatial datasets including information on physical environment.

*Use of model to determine:* To examined whether neighbourhood exposure to fast-food outlets and physical activity facilities were associated with adiposity in UK adults

*Type of model:* Regression model

*Models and parameters used in the study:* Multilevel, multiple linear regression models with random intercepts and random coefficients were used for the main exposure to estimate independent associations between each environmental exposure and each adiposity outcome, accounting for the nesting of individuals within assessment centres. Initially the model was adjusted only for age and sex (model 0), then for likely demographic confounders (age, sex, ethnicity, area deprivation, and urbanicity; model 1), then further adjusted for individual level socioeconomic characteristics (income, education, and employment status; model 2) and, finally, for the non-exposure environmental feature (proximity to fast food or density of physical activity facilities) and neighbourhood residential density (model 3). As well as adjusting for potential confounding by sex and income, the models were also tested fully adjusted for effect modification by these variables. The results were reported in stratified form where models with interaction terms for sex or income were statistically different from those without (likelihood ratio test p<0.05). This study also estimated the same models using height as a negative control outcome.

1. **Multivariate logistic regression**

*Model description:* Multiple logistic regression is distinguished from multiple linear regression in that the outcome variable (dependent variables) is dichotomous (e.g., diseased or not diseased). Its aim is the same as that of all model-building techniques: to derive the best-fitting, most parsimonious (smallest or most efficient), and biologically reasonable model to describe the relationship between an outcome and a set of predictors. Here, the independent variables are called covariates. Importantly, in multiple logistic regression, the predictor variables may be of any data level (categorical, ordinal, or continuous). A major use of this technique is to examine a series of predictor variables to determine those that best predict a certain outcome.

*Title of the study:* Development and validation of risk prediction model for venous thromboembolism in postpartum women: multinational cohort study

*Link to the study:*  <https://doi.org/10.1136/bmj.i6253>

*Study design:* Cohort study

*Domain:* Medical care

*Data sources used:* The Clinical Practice Research Datalink (CPRD) is a large, longitudinal UK primary care linked database that covers 6% of the population was used as a derivation cohort and Swedish birth registry as a validation cohort.

*Use of model to determine:* To develop and validate a risk prediction model for venous thromboembolism in the first six weeks after delivery (early postpartum).

*Type of model:* Classification model

*Models and parameters used in the study:* The occurrence of venous thromboembolism during the first six weeks postpartum was treated as a binary outcome measure. For each of the 22 candidate predictors, we used a univariable logistic regression model to calculate the unadjusted odds ratio. For derivation of the risk prediction model, initially all candidate predictors in a multivariable logistic regression model were included. A clustering term was fitted to take account of consecutive pregnancies within women during the study period and used fractional polynomials to model potential non-linear relations between outcome and continuous predictors. All variables are coded as binary (0 or 1 for absence or presence of a risk factor), except for age, body mass index (BMI), and birth weight. These three variables were transformed on the basis of fractional polynomial regression (first degree) analysis. The value −9.103 is the intercept, and other numbers are the estimated regression coefficients for the predictors, which indicate their mutually adjusted relative contribution to the outcome risk. The regression coefficients represent the log odds ratio for a change of 1 unit in the corresponding predictor. The predicted risk of VTE=1/1+e−riskscore.

1. **Multivariable hierarchical modified Poisson regression**

*Model description:* Modified Poisson regression, which combines a log Poisson regression model with robust variance estimation, is a useful alternative to log binomial regression for estimating relative risks.

*Title of the study:* Association Between Prompt Defibrillation and Epinephrine Treatment With Long-Term Survival After In-Hospital Cardiac Arrest

*Link to the study:* <https://doi.org/10.1161/CIRCULATIONAHA.117.030488>

*Study design:* Cohort study

*Domain:* Medical care

*Data sources used:* Data from American Heart Association’s GWTG (Get With The Guidelines)-Resuscitation registry, which is a large, prospective, quality-improvement registry of IHCAs (In-Hospital Cardiac Arrests) linked with inpatient files of Medicare.

*Use of model to determine:* To examine long-term survival according to the promptness of defibrillation and epinephrine administration in patients with an IHCA resulting from shockable and nonshockable rhythms, respectively.

*Type of model:* Regression model

*Models and parameters used in the study:* To assess the associations between prompt treatment and long-term survival for each rhythm type, hierarchical multivariable modified Poisson regression models were constructed. Modified Poisson regression was used to correct for overestimation of estimates of effect observed with odds ratios when the outcome rate exceeds 10%.Instead, Poisson models yield relative risk estimates obtained from a Poisson distribution. Moreover, these models were hierarchical models, with site as a random effect and patient-level factors as fixed effects. Specifically, they modeled as fixed effects age, sex, race, time to start of cardiopulmonary resuscitation, location of cardiac arrest, and different coexisting conditions and events present within 24 hours before the cardiac arrest. In addition, the models were adjusted for interventions in place at the time of cardiac arrest, day of the week, and calendar of year admission of cardiac arrest.

1. **Cox regression model**

*Model description:* The **Cox proportional-hazards model** is essentially a regression model commonly used statistical in medical research for investigating the association between the survival time of patients and one or more predictor variables.

*Title of the study:* Variation in cardiovascular disease care: an Australian cohort study on sex differences in receipt of coronary procedures

*Link to the study:* <http://dx.doi.org/10.1136/bmjopen-2018-026507>

*Domain:* Medical care

*Study design:* Prospective cohort study

*Data sources used:* A population cohort data linked with hospital data and a death registry.

*Use of model to determine:* Time dependent variation

*Type of model:* Regression model

*Models and parameters used in the study:* Cox proportional hazard regression was used to model the association between sex and receipt of coronary procedures. For each analysis, participants contributed person-years from the date of index admission for AMI or angina until either the specified outcome of interest, death from any cause or end of follow-up (30 June 2016), whichever was the earliest, to a maximum of one calendar year. Data from patients in the angina sample were also censored if they were subsequently admitted with AMI. Proportional hazards assumption was tested, with the p-value set a priori to p<0.01. All analyses were conducted separately for patients whose index admission was for AMI, and for those whose index admission was for angina. Patients presenting concurrently with AMI and angina were included in the AMI sample.
For each outcome, we calculated crude incidence rates separately for men and women, then ran a series of Cox regression models to estimate HRs in relation to sex. Model 1 was adjusted for age (5-year age categories from 45 to 54 years through to ≥80 years). Model 2 was adjusted for age and sociodemographic variables (country of birth, region of residence, highest qualification, private health insurance and marital status). Model 3 was further adjusted for additional baseline health characteristics (obesity, physical functioning and psychological distress). Participants with missing values for covariates were included in the models, with missing coded as a separate category.

1. **LASSO (Least Absolute Shrinkage and Selection Operator) model**

*Model description:* LASSO model is a [regression analysis](https://en.wikipedia.org/wiki/Regression_analysis) method that performs both [variable selection](https://en.wikipedia.org/wiki/Variable_selection) and [regularization](https://en.wikipedia.org/wiki/Regularization_(mathematics)) in order to enhance the prediction accuracy and interpretability of the [statistical model](https://en.wikipedia.org/wiki/Statistical_model) it produces.

*Title of the study:* Long-term chronic diseases and crash responsibility: A record linkage study

*Link to the study:* <https://doi.org/10.1016/j.aap.2014.05.001>

*Domain:* Health status monitoring

*Study design:* A case-control study

*Data sources used:* Data from three French national databases were extracted and matched: the national healthcare insurance database, police reports and the national police database of injurious crashes.

*Use of model to determine:* To assess the population impact of chronic conditions on the risk of road traffic crashes

*Type of model:* Regression model

*Models and parameters used in the study:* A single model adjusted for crash-related and socio-demographic factors, including all the 299 long-term diseases as covariates, using the Lasso (least absolute shrinkage and selection operator) method was fitted (Avalos et al., 2012; Tibshirani, 1996). Adjustment variables (age, gender, socioeconomic category, year, season, day, time and location of crash, vehicle type, injury severity, blood alcohol concentration and exposure to level 2 and 3 medicines)were forced into the model; the proper amount of shrinkage of the long-term disease covariates was estimated using the Akaike information criterion (AIC) and was corrected for bias. One limitation of the Lasso method is that with a proper amount of shrinkage relevant covariates are retained, but so too are a few additional irrelevant ones (though, typically, their estimates are small). Different procedures have been proposed in the literature to address this particular problem, such as those based on bootstrap-enhanced Lasso (Avaloset al., 2012; Bach, 2008; Bunea et al., 2011). Thus, to reduce the false discovery rate, only chronic condition covariates chosen more frequently by the Lasso over the 5000 bootstrap samples were selected and investigated further. The threshold frequency (75% of the bootstrapped models) was also chosen by AIC. In order to control for multiple medical conditions, a variable was introduced in the multivariable analyses, representing all other chronic diseases than the ones specifically identified. We used the R package glmnet (R Development Core Team, 2011). We also fitted 299 separate logistic regression models, disease by disease, using conventional maximum likelihood adjusting for crash-related and socio-demographic factors. Analyses were performed with and without Bonferroni correction for multiple testing, in order to compare results from the Lasso method with a conventional modeling strategy.

1. **Generalized Estimating Equation (GEE) models**

# *Model description:*  GEE is used to [estimate](https://en.wikipedia.org/wiki/Estimator) the parameters of a [generalized linear model](https://en.wikipedia.org/wiki/Generalized_linear_model) with a possible unknown [correlation](https://en.wikipedia.org/wiki/Correlation_and_dependence) between outcomes. Parameter estimates from the GEE are [consistent](https://en.wikipedia.org/wiki/Consistent_estimator) even when the [covariance](https://en.wikipedia.org/wiki/Covariance) structure is misspecified, under mild regularity conditions. The focus of the GEE is on estimating the average response over the population ("population-averaged" effects) rather than the [regression](https://en.wikipedia.org/wiki/Regression_analysis) parameters that would enable prediction of the effect of changing one or more covariates on a given individual.

# *Title of the study:* An analysis of weight perception and physical activity and dietary behaviours among youth in the COMPASS study

*Link to the study:* <https://doi.org/10.1016/j.ssmph.2016.10.016>

*Domain:* Health status monitoring

*Study design:* Cohort study

*Data sources used:* This study used 2-year linked data of 19,322 grade 9-12 students from Year 2 (Y_2_:2013-2014) and 3 (Y_3_:2014-2015) of the COMPASS study.

*Use of model to determine:* to examine how weight perception influences physical activity (PA) and diet among youth.

*Models and parameters used in the study:* Generalized Estimating Equations (GEE) models were used to test the effect of Y3 weight perception (underweight, overweight, “about right”) on the various Y3 outcome measures of PA and dietary behaviours, adjusting for Y3 covariates (grade, race/ethnicity, weekly spending money, school area median household income) and the Y2 outcome health behaviour. Models were stratified by gender and BMI status. In other words, the models included Y3 data for the predictor, covariate, and outcome measures, and adjusted for the outcome measure from Y2 data, in order to strengthen inferences. The GEE model is an extension of generalized linear models to correlated data, simply modelling the mean response and treating covariance as nuisance. It produces consistent estimates for regression parameters and can be used for continuous, categorical (including binary), and ordinal measurements. In our analyses, we specified identity link function for continuous outcomes, logit for binary outcomes and cumulative logit for ordinal outcomes. Schools were included in the models as clusters to take account of within-school correlation. Squared root transformation was used for continuous outcome variables to meet model assumptions.

1. **Inverse probability weighting (IPW) methods**

*Model description:* Inverse probability weighting is a statistical technique for calculating statistics standardized to a [pseudo-population](https://en.wikipedia.org/w/index.php?title=Pseudo-population&action=edit&redlink=1) different from that in which the data was collected. Study designs with a disparate sampling population and population of target inference (target population) are common in application.

# *Title of the study:* The Impact of Cardiac Rehabilitation (CR) and Secondary Prevention Programs on 12-Month Clinical Outcomes: A Linked Data Analysis

*Link to the study:* <https://doi.org/10.1016/j.hlc.2019.03.015>

*Domain:* Medical care

*Study design:* Retrospective cohort design

*Data sources used:* Cardiac Rehabilitation databases were linked to hospital administrative datasets

*Use of model to determine:* to determine if CR attendance impacts on cardiovascular readmission, morbidity and mortality.

*Models and parameters used in the study:* An inverse probability weighting (IPW) model was used to account for selection bias and unequal probabilities of those patients attending or not attending CR. Factors in the IPW model included age, gender, primary diagnosis, Charlson Index, prior HF, coronary disease, AF, revascularization, malignancy and social factors measured by the IRSAD. The IRSAD measures high and low income, degree of house mortgage, size of home, educational level, qualifications or none, employed as a professional, a manager, low skilled worker, machinery operator or labourer, high rent, number of cars or none, overcrowding, divorced, low rent, disability, unemployed single parent family, no internet access and jobless parents from the Australian Census. Each ED presentation resulting in a separation was considered a single hospitalization and each cardiac admission was counted as a single separation, with admissions involving transfer(s) merged as one. Readmission within 24 hours was not counted as a new event. For assessment of associations between CR attendance and cardiovascular events (cardiovascular readmission, death, new/re-MI, HF, AF or stroke), an analysis confined to those patients referred to CR was undertaken. The balance of the IPW weighted population is presented in Table 1. Further, cardiovascular events occurring prior to 70 days from discharge were removed from the analysis and outcomes were measured post CR program within 12 months. Associations with CR attendance and outcomes were measured by Cox proportional hazard models applied to the IPW population and stratified by primary cardiac diagnosis, referring hospital, Charlson Index and adjusted for age, gender and socioeconomic status. The proportional hazards assumption was assessed and found to be valid.

1. **Blinder-Oaxaca decomposition method**

*Model description:* The Blinder–Oaxaca decomposition is a statistical method that explains the difference in the [means](https://en.wikipedia.org/wiki/Mean) of a [dependent variable](https://en.wikipedia.org/wiki/Dependent_variable) between two groups by decomposing the gap into that part that is due to differences in the mean values of the independent variable within the groups, on the one hand, and group differences in the effects of the independent variable, on the other hand.

# Title of the study: Activity limitations predict health care expenditures in the general population in Belgium

*Link to the study:* <https://doi.org/10.1186/s12889-015-1607-7>

*Study design:* Retrospective cohort design

*Domain:* Health status monitoring

*Data sources used:* Data from the Belgian Health Interview Survey 2008 were linked with data from the compulsory national health insurance (n = 7,286).

*Use of model to determine:* The predictive value of the GALI (Global Activity Limitation Indicator) on health care expenditures in relation to the presence of chronic conditions.

*Models and parameters used in the study:* To study the factors contributing to the difference in health expenditure between persons with and without activity limitations, the Blinder-Oaxaca decomposition method was used. Although multivariate regression models are suitable to address differences in the importance of individual factors, the Blinder-Oaxaca technique demonstrates the relative importance of each predictor. The decomposition illustrates the fraction of the gap in health care expenditures that is attributable to group differences in the magnitude of the determinants (the explained or prevalence component) and to group differences in the effects of these determinants (the unexplained or impact component). The Blinder-Oaxaca decomposition method is particularly useful to study differences in health care expenditures between two groups, but it has also been used in studies in which the contribution of both the prevalence and the impact of determinants to explain differences between groups was investigated for other health outcomes.

1. **Markov modelling**

*Model description:* In [probability theory](https://en.wikipedia.org/wiki/Probability_theory), a Markov model is a [stochastic model](https://en.wikipedia.org/wiki/Stochastic_model) used to [model](https://en.wikipedia.org/wiki/Mathematical_model) randomly changing systems. It is assumed that future states depend only on the current state, not on the events that occurred before it (that is, it assumes the [Markov property](https://en.wikipedia.org/wiki/Markov_property)). Generally, this assumption enables reasoning and computation with the model that would otherwise be [intractable](https://en.wikipedia.org/wiki/Intractability_(complexity)).

*Title of the study:* Using electronic health records to predict costs and outcomes in stable coronary artery disease

*Link to the study:* <http://dx.doi.org/10.1136/heartjnl-2015-308850>

*Domain:* Medical care

*Study design:* Retrospective cohort

*Data sources used:* The analysis was based on 94 966 patients with stable-CAD (Coronary Artery Disease) in England between 2001 and 2010, identified in four prospectively collected, linked EHR sources.

*Use of model to determine:* To predict lifetime costs and health outcomes of patients with stable coronary artery disease (stable-CAD) stratified by their risk of future cardiovascular events, and to evaluate the cost-effectiveness of treatments targeted at these populations.

*Type of model:* Predictive model

*Models and parameters used in the study:* A state transition model (shown in figure 1) was developed to capture the natural history of patients with stable-CAD. The structure of the model was determined with reference to both previous models in CVD13 and expert clinical advice. All patients entered the model in the stable-CAD state and progressed through the model until they experienced either CVD or non-CVD mortality. The time horizon of the model was, therefore, the patient’s remaining lifetime. The model captured time varying and age-dependent risks, costs and health-related quality of life (HRQoL) in 90-day segments. Costs and HRQoL were attached to model states and, in order to stratify by patients’ baseline risk, adjusted for patient covariates at baseline as well as for age and for time elapsed following non-fatal events. Model predicted costs, life years and QALYs were discounted at 3.5% per annum in keeping with the guidelines in England.18 While only first occurrences of non-fatal CVD events were explicitly modelled, further non-fatal events were implicitly captured in the time varying risk, cost and HRQoL estimates.

**2. Supervised Machine Learning Techniques (N = 10)**

1. **Linear Discriminant Analysis (LDA)**

*Model description:* Linear discriminant analysis (LDA), normal discriminant analysis (NDA), or discriminant function analysis is a generalization of Fisher's linear discriminant, a method used in [statistics](https://en.wikipedia.org/wiki/Statistics), [pattern recognition](https://en.wikipedia.org/wiki/Pattern_recognition), and [machine learning](https://en.wikipedia.org/wiki/Machine_learning) to find a [linear combination](https://en.wikipedia.org/wiki/Linear_combination) of [features](https://en.wikipedia.org/wiki/Features_(pattern_recognition)) that characterizes or separates two or more classes of objects or events. The resulting combination may be used as a [linear classifier](https://en.wikipedia.org/wiki/Linear_classifier), or, more commonly, for [dimensionality reduction](https://en.wikipedia.org/wiki/Dimensionality_reduction) before later [classification](https://en.wikipedia.org/wiki/Statistical_classification).

# *Title of the study:* Optimizing Machine Learning Methods to Improve Predictive Models of Alzheimer’s Disease

*Link to the study:*  https://dx.doi.org/[10.3233/JAD-190262](https://dx.doi.org/10.3233%2FJAD-190262)

*Study design:* cohort study

*Domain:* Clinical care

*Data sources used:* Data from an ongoing cohort study

*Use of model to determine:* To classify cognitively normal (CN) individuals from Alzheimer’s disease (AD) and to predict longitudinal outcome in participants with mild cognitive impairment (MCI

*Type of model:* Predictive model

*Models and parameters used in the study:* In this study, four features set and six machine-learning methods (decision trees, support vector machines, K-nearest neighbor, ensemble linear discriminant, boosted trees, and random forests) were used to classify participants with normal cognition from participants with AD. Subsequently the model with best classification performance was used for predicting clinical outcome of MCI participants.

1. **Partial least square discriminant analysis model**

*Model description:* Partial least squares regression (PLS regression) is a [statistical](https://en.wikipedia.org/wiki/Statistics) method that bears some relation to [principal components regression](https://en.wikipedia.org/wiki/Principal_component_regression); instead of finding [hyperplanes](https://en.wikipedia.org/wiki/Hyperplane) of maximum [variance](https://en.wikipedia.org/wiki/Variance) between the response and independent variables, it finds a [linear regression](https://en.wikipedia.org/wiki/Linear_regression) model by projecting the [predicted variables](https://en.wikipedia.org/wiki/Predicted_variable) and the [observable variables](https://en.wikipedia.org/wiki/Observable_variable) to a new space. Because both the *X* and *Y* data are projected to new spaces, the PLS family of methods are known as bilinear factor models.

# *Title of the study:* An exploration of mortality risk factors in non-severe pneumonia in children using clinical data from Kenya

*Link to the study:* <https://doi.org/10.1186/s12916-017-0963-9>

*Domain:* Medical care

*Study design:* Retrospective cohort study

*Data sources used:* Hospital medical admission and discharge reports and laboratory data

*Use of model to determine:* To identify factors that best discriminate inpatient mortality risk in non-severe pneumonia and explore whether these factors offer any added benefit over the current criteria used to identify children with pneumonia requiring inpatient care.

*Type of model:*

*Models and parameters used in the study:* In this study, the machine learning models used in analysis were partial least squares - discriminant analysis (PLS-DA), random forests (RFs), support vector machines (SVMs) and elastic nets. Model validation as checked by employing a 10-fold internal cross validation on two thirds of the data. The remaining one third of the data was used as the validation set. The selection of critical parameters for each of these modelling techniques was auto-determined by the R caret train function by choosing the tuning parameters that produced the highest values of receiver operating characteristic (ROC) curves where a grid search crossvalidation was applied.

1. **Decision tree learning**

*Model description:* Decision tree learning is one of the predictive modelling approaches used in [statistics](https://en.wikipedia.org/wiki/Statistics), [data mining](https://en.wikipedia.org/wiki/Data_mining) and [machine learning](https://en.wikipedia.org/wiki/Machine_learning). It uses a [decision tree](https://en.wikipedia.org/wiki/Decision_tree) (as a [predictive model](https://en.wikipedia.org/wiki/Predictive_modelling)) to go from observations about an item (represented in the branches) to conclusions about the item's target value (represented in the leaves). Tree models where the target variable can take a discrete set of values are called classification trees; in these tree structures, [leaves](https://en.wikipedia.org/wiki/Leaf_node) represent class labels and branches represent [conjunctions](https://en.wikipedia.org/wiki/Logical_conjunction) of features that lead to those class labels. Decision trees where the target variable can take continuous values (typically [real numbers](https://en.wikipedia.org/wiki/Real_numbers)) are called regression trees. Decision trees are among the most popular machine learning algorithms given their intelligibility and simplicity.

# *Title of the study:* Application of Machine Learning to Predict Dietary Lapses During Weight Loss

*Link to the study:* [https://doi.org/10.1177/1932296818775757](https://doi.org/10.1177%2F1932296818775757)

*Domain:* Health status monitoring

*Study design:* An online survey

*Data sources used:* An online Weight Watchers program (i.e., an evidence-based program) to loss the weight

*Use of model to determine:* prediction of dietary lapses during weight loss

*Type of model:* Predictive model

*Models and parameters used in the study:* In this study, the optimal group model was identified using ensemble methods (e.g., combining weighted vote of predictions from Random Forest, Logit. Boost, Bagging, Random Subspace, Bayes Net). Cost-sensitive methods were used by incorporating a cost matrix (e.g., a matrix of penalties for misclassification) into each decision tree. Cost sensitive penalties were selected based on a balance of sensitivity and specificity (e.g., highest possible sensitivity while maintaining adequate specificity).

1. **Random forest**

*Model description:* Random forests or random decision forests are an [ensemble learning](https://en.wikipedia.org/wiki/Ensemble_learning) method for [classification](https://en.wikipedia.org/wiki/Statistical_classification), [regression](https://en.wikipedia.org/wiki/Regression_analysis) and other tasks that operate by constructing a multitude of [decision trees](https://en.wikipedia.org/wiki/Decision_tree_learning) at training time and outputting the class that is the [mode](https://en.wikipedia.org/wiki/Mode_(statistics)) of the classes (classification) or mean prediction (regression) of the individual trees. Random decision forests correct for decision trees habit of [overfitting](https://en.wikipedia.org/wiki/Overfitting) to their [training set](https://en.wikipedia.org/wiki/Test_set).

# *Title of the study:* Machine learning models in breast cancer survival prediction

*Link to the study:*  <https://pubmed.ncbi.nlm.nih.gov/26409558/>

*Domain:* Medical care

*Study design:* Cohort study

*Data sources used:* A dataset with eight attributes that include the records of 900 patients in which 876 patients (97.3%) and 24 (2.7%) patients were females and males respectively

Use of model to determine: To propose a rule-based classification method with machine learning techniques for the prediction of different types of Breast cancer survival.

*Type of model:* Prediction model

*Models and parameters used in the study:* In this study, following models were used for the prediction of breast cancer survival Naive Bayes (NB), Trees Random Forest (TRF), 1-Nearest Neighbor (1NN), AdaBoost (AD), Support Vector Machine (SVM), RBF Network (RBFN), and Multilayer Perceptron (MLP) machine learning techniques with 10-cross fold technique. The performance of machine learning techniques were evaluated with accuracy, precision, sensitivity, specificity, and area under ROC curve.

1. **Gradient Boosting Classifier (GBC)**

*Model description:* Gradient boosting is a [machine learning](https://en.wikipedia.org/wiki/Machine_learning) technique for [regression](https://en.wikipedia.org/wiki/Regression_(machine_learning)) and [classification](https://en.wikipedia.org/wiki/Classification_(machine_learning)) problems, which produces a prediction model in the form of an [ensemble](https://en.wikipedia.org/wiki/Ensemble_learning) of weak prediction models, typically [decision trees](https://en.wikipedia.org/wiki/Decision_tree_learning). It builds the model in a stage-wise fashion like other [boosting](https://en.wikipedia.org/wiki/Boosting_(machine_learning)) methods do, and it generalizes them by allowing optimization of an arbitrary [differentiable](https://en.wikipedia.org/wiki/Differentiable_function) [loss function](https://en.wikipedia.org/wiki/Loss_function).

*Title of the study:* Machine learning for characterizing risk of type 2 diabetes mellitus in a rural Chinese population: the Henan Rural Cohort Study

*Link to the study:* <https://doi.org/10.1038/s41598-020-61123-x>

*Domain:* Health status monitoring

*Study design:* Cohort study

*Data sources used:* Data on socio-demographic characteristics, information on physical examination, and laboratory test data

*Use of model to determine:* To test the ability of machine learning algorithms for predicting risk of type 2 diabetes mellitus (T2DM)

*Type of model:* Predictive model

*Models and parameters used in the study:* In this study, risk assessment models for T2DM were developed using six machine learning algorithms, including logistic regression (LR), classification and regression tree (CART), artificial neural networks (ANN), support vector machine (SVM), random forest (RF) and gradient boosting machine (GBM). The model performance was measured in an area under the receiver operating characteristic curve, sensitivity, specificity, positive predictive value, negative predictive value and area under precision recall curve. The importance of variables was identified based on each classifier and the shapley additive explanations approach. Using all available variables, all models for predicting risk of T2DM demonstrated strong predictive performance, with AUCs ranging between 0.811 and 0.872 using laboratory data and from 0.767 to 0.817 without laboratory data. Among them, the GBM model performed best (AUC: 0.872 with laboratory data and 0.817 without laboratory data). Performance of models plateaued when introduced 30 variables to each model except CART model. Among the top-10 variables across all methods were sweet flavor, urine glucose, age, heart rate, creatinine, waist circumference, uric acid, pulse pressure, insulin, and hypertension. New important risk factors (urinary indicators, sweet flavor) were not found in previous risk prediction methods, but determined by machine learning in our study. Through the results, machine learning methods showed competence in predicting risk of T2DM, leading to greater insights on disease risk factors with no priori assumption of causality.

1. **k-nearest neighbours/k-means**

*Model description:* In [pattern recognition](https://en.wikipedia.org/wiki/Pattern_recognition), the *k*-nearest neighbors algorithm (*k*-NN) is a [non-parametric](https://en.wikipedia.org/wiki/Non-parametric_statistics) method proposed by [Thomas Cover](https://en.wikipedia.org/wiki/Thomas_M._Cover) used for [classification](https://en.wikipedia.org/wiki/Statistical_classification) and [regression](https://en.wikipedia.org/wiki/Regression_analysis). In both cases, the input consists of the *k* closest training examples in the [feature space](https://en.wikipedia.org/wiki/Feature_space). The output depends on whether *k*-NN is used for classification or regression:

- In *k-NN classification*, the output is a class membership. An object is classified by a plurality vote of its neighbors, with the object being assigned to the class most common among its *k* nearest neighbors (*k* is a positive [integer](https://en.wikipedia.org/wiki/Integer), typically small). If *k* = 1, then the object is simply assigned to the class of that single nearest neighbor.
- In *k-NN regression*, the output is the property value for the object. This value is the average of the values of *k* nearest neighbors.

*Title of the study:* Machine Learning With K-Means Dimensional Reduction for Predicting Survival Outcomes in Patients With Breast Cancer

*Link to the study:* [https://doi.org/10.1177/1176935118810215](https://doi.org/10.1177%2F1176935118810215)

*Domain:* Health care

*Study design:* Prospective cohort study

*Data sources used:* Clinicopathological and genomic data

*Use of model to determine:* to integrate multiple clinicopathological and genomic factors with dimensional reduction across machine learning algorithms to compare survival predictions.

*Models and parameters used in the study:* In this study, to predict survival outcome, a total of 27 features (including indicator variables) from the 18 clinicopathological features mentioned above and 1 genomic feature were used to construct the models. We trained a series of nonlinear machine learning methods with 10-fold cross-validation of the training set upon 10 random training/validation splits using Gradient Boosting (R package *xgboost*), Random Forest (R package *random Forest*), SVM with a radial basis (SVM, R package *svm*), and ANN (R package *nnet*). The 10 random training/validation splits included the same patients in each set as those for K-means clustering above. For each split, 80% of the analytic cohort were randomly selected as our training dataset. Model performance was examined in the remaining 20% validation dataset, by estimating ROC, accuracy, and CS.

1. **Support Vector Machine**

*Model description:* In [machine learning](https://en.wikipedia.org/wiki/Machine_learning), support-vector machines (SVMs, also support-vector networks) are [supervised learning](https://en.wikipedia.org/wiki/Supervised_learning) models with associated learning [algorithms](https://en.wikipedia.org/wiki/Algorithm) that analyze data used for [classification](https://en.wikipedia.org/wiki/Statistical_classification) and [regression analysis](https://en.wikipedia.org/wiki/Regression_analysis).

*Title of the study:* A machine learning-based framework to identify type 2 diabetes through electronic health records

*Link to the study:* <https://doi.org/10.1016/j.ijmedinf.2016.09.014>

*Domain:* Health status monitoring

*Study design:* Cohort study

*Data sources used:* Electronic health records

*Use of model to determine:*  to develop a semi-automated framework based on machine learning as a pilot study to liberalize filtering criteria to improve recall rate with a keeping of low false positive rate

*Models and parameters used in the study:* In this study, several widely-used classification model such as [k-Nearest-Neighbors](https://www.sciencedirect.com/topics/nursing-and-health-professions/k-nearest-neighbor) (kNN), [Naïve Bayes](https://www.sciencedirect.com/topics/nursing-and-health-professions/bayesian-learning) (NB), [Decision Tree](https://www.sciencedirect.com/topics/computer-science/decision-trees) (J48), [Random Forest](https://www.sciencedirect.com/topics/computer-science/random-decision-forest) (RF), Support Vector Machine (SVM) and [Logistic Regression](https://www.sciencedirect.com/topics/nursing-and-health-professions/logistic-regression-analysis) (LR) to model patterns of cases and controls based on our extracted features and then use the models to test the ability of our extracted features on identifications of T2DM subjects.

1. **Neural networks**

*Model description:* These are the systems modeled after the human brain, mimicking the ways we learn and make decisions. These networks consist of input and output layers, as well as hidden layers, similar to the neural networks in our brains.

*Title of the study:* Machine learning approaches to the social determinants of health in the health and retirement study

*Link to the study:* <https://doi.org/10.1016/j.ssmph.2017.11.008>

*Domain:* social determinants

*Study design:* Retrospective cohort

*Data sources used:* Health and retirement study database

*Use of model to determine:* To investigate how machine learning may add to our understanding of social determinants of health using data from the Health and Retirement Study.

*Models and parameters used in the study:* To assess different machine learning methods’ ability to predict the biomarkers of interest, we first considered two OLS (Ordinary Least Square) regression models. The first was minimal and included gender, age, and age squared. The second was based on current understanding of social determinants of health, particularly that education and economic position have demonstrated associations with health. This theory-based model was parsimonious and included, as linear variables, household income, household wealth, and two binary variables indicating a high school-level education and less than a high school-level education, in addition to the parameters in the minimal model.

We next consider four machine learning algorithms: repeated linear regressions - akin to genome-wide association studies (GWAS), penalized linear regressions ([Hastie, 2009](https://www.sciencedirect.com/science/article/pii/S2352827317302331?via%3Dihub#bib12)), random forests ([Breiman, 2001](https://www.sciencedirect.com/science/article/pii/S2352827317302331?via%3Dihub#bib4)), and [neural networks](https://www.sciencedirect.com/topics/social-sciences/neural-network) ([Kriesel, 2007](https://www.sciencedirect.com/science/article/pii/S2352827317302331?via%3Dihub#bib19)). These cover parametric and nonparametic approaches, with varying abilities to account for nonlinearity. While it is not possible to consider all machine learning algorithms, in addition to the broad coverage offered by these algorithms, all have been used in the medical literature ([Patel et al., 2010](https://www.sciencedirect.com/science/article/pii/S2352827317302331?via%3Dihub#bib22), [Rehkopf and Laraia, 2011](https://www.sciencedirect.com/science/article/pii/S2352827317302331?via%3Dihub#bib30), [Horvath, 2013](https://www.sciencedirect.com/science/article/pii/S2352827317302331?via%3Dihub#bib16), [Kapetanovic et al., 2004](https://www.sciencedirect.com/science/article/pii/S2352827317302331?via%3Dihub#bib18), [Sato et al., 2005](https://www.sciencedirect.com/science/article/pii/S2352827317302331?via%3Dihub#bib33), [Goldstein et al., 2010](https://www.sciencedirect.com/science/article/pii/S2352827317302331?via%3Dihub#bib11)) and penalized regressions and random forests are particularly commonly-taught methods ([Hastie et al., 2009](https://www.sciencedirect.com/science/article/pii/S2352827317302331?via%3Dihub#bib12), [Bishop, 2006](https://www.sciencedirect.com/science/article/pii/S2352827317302331?via%3Dihub#bib3)). These four also offer some prospect for interpretation rather than being completely “black box” approaches.

1. **Hierarchical clustering**

*Model description:* n [data mining](https://en.wikipedia.org/wiki/Data_mining) and [statistics](https://en.wikipedia.org/wiki/Statistics), hierarchical clustering (also called hierarchical cluster analysis or HCA) is a method of [cluster analysis](https://en.wikipedia.org/wiki/Cluster_analysis) which seeks to build a [hierarchy](https://en.wikipedia.org/wiki/Hierarchy) of clusters. Strategies for hierarchical clustering generally fall into two types:[^[1]^](https://en.wikipedia.org/wiki/Hierarchical_clustering#cite_note-clusteringMethods-1)

- Agglomerative: This is a "[bottom-up](https://en.wikipedia.org/wiki/Top-down_and_bottom-up_design)" approach: each observation starts in its own cluster, and pairs of clusters are merged as one moves up the hierarchy.
- Divisive: This is a "[top-down](https://en.wikipedia.org/wiki/Top-down_and_bottom-up_design)" approach: all observations start in one cluster, and splits are performed recursively as one moves down the hierarchy.

# *Title of the study:* Novel subgroups of adult-onset diabetes and their association with outcomes: a data-driven cluster analysis of six variables

*Link to the study:* <https://doi.org/10.1016/S2213-8587(18)30051-2>

*Domain:* Health status monitoring

*Study design:* A data driven cluster analysis

*Data sources used:* Diabetes registry

*Use of model to determine:*

*Models and parameters used in the study:* In this study, a data-driven cluster analysis (k-means and hierarchical clustering) in patients with newly diagnosed diabetes (n=8980) from the Swedish All New Diabetics in Scania cohort. Clusters were based on six variables (glutamate decarboxylase antibodies, age at diagnosis, BMI, HbA1c, and homoeostatic model assessment 2 estimates of β-cell function and insulin resistance), and were related to prospective data from patient records on development of complications and prescription of medication.

1. **XGBoost**

*Model description:* XGBoost is an [open-source](https://en.wikipedia.org/wiki/Open-source_software) [software library](https://en.wikipedia.org/wiki/Library_(computing)) that provides a machine learning method of regression and classification using ensemble learning with gradient tree boosting (GTB). This software provides a [gradient boosting](https://en.wikipedia.org/wiki/Gradient_boosting) framework for [C++](https://en.wikipedia.org/wiki/C%2B%2B), [Java](https://en.wikipedia.org/wiki/Java_(programming_language)), [Python](https://en.wikipedia.org/wiki/Python_(programming_language)), [R](https://en.wikipedia.org/wiki/R_(programming_language)), [Julia](https://en.wikipedia.org/wiki/Julia_(programming_language)), [Perl](https://en.wikipedia.org/wiki/Perl_(programming_language)), and [Scala](https://en.wikipedia.org/wiki/Scala_(programming_language)). It works on [Linux](https://en.wikipedia.org/wiki/Linux), [Windows](https://en.wikipedia.org/wiki/Windows), and [macOS](https://en.wikipedia.org/wiki/MacOS). From the project description, it aims to provide a "Scalable, Portable and Distributed Gradient Boosting (GBM, GBRT, GBDT) Library". It runs on a single machine, as well as the distributed processing frameworks [Apache Hadoop](https://en.wikipedia.org/wiki/Apache_Hadoop), [Apache Spark](https://en.wikipedia.org/wiki/Apache_Spark), and [Apache Flink](https://en.wikipedia.org/wiki/Apache_Flink).

## *Title of the study:* Prediction of Glucose Metabolism Disorder Risk Using a Machine Learning Algorithm: Pilot Study

*Link to the study:* <https://pubmed.ncbi.nlm.nih.gov/30478026/>

*Domain:* Health status monitoring

*Study design:* Retrospective cohort study

*Data sources used:* Medical records

*Use of model to determine:* To predict the risk of developing diabetes or GMD (Glucose Metabolism Disorder) using data from thousands of OGTTs (Oral Glucose Tolerance Test) and a machine learning technique (XGBoost)

*Models and parameters used in the study:* XGBoost is open-source software that provides a machine learning method of regression and classification using ensemble learning with gradient tree boosting (GTB). For each study, to apply supervised machine learning methods, the required label data was prepared. If a subject was diagnosed with diabetes or GMD at least once during the period, then that subject’s data obtained in previous trials were classified into the risk group (y=1). After data processing, 13,581 and 6760 OGTTs were analyzed for study 1 and study 2, respectively. For each study, a randomly chosen subset representing 80% of the data was used for training 9 classification models and the remaining 20% was used for evaluating the models. Three classification models, A to C, used XGBoost with various input variables, some including OGTT data. The other 6 classification models, D to I, used LR for comparison.
